# Addressing the Challenges in the Diagnosis and Management of Pediatric Wilson’s Disease—Case Report and Literature Review

**DOI:** 10.3390/medicina59040786

**Published:** 2023-04-18

**Authors:** Irene Maria Ungureanu, Mara Ioana Iesanu, Catalin Boboc, Vlad Cosoreanu, Lorena Vatra, Anna Kadar, Evelina Nicoleta Ignat, Felicia Galos

**Affiliations:** 1 Department of Pediatrics, Marie Curie Emergency Children’s Hospital, 041451 Bucharest, Romania; 2 Department of Physiology, Carol Davila University of Medicine and Pharmacy, 020021 Bucharest, Romania; 3 Department of Pediatric Surgery, Marie Curie Emergency Children’s Hospital, 041451 Bucharest, Romania; 4 Department of Pediatric Surgery, Carol Davila University of Medicine and Pharmacy, 020021 Bucharest, Romania; 5 Department of Pathology, Colentina Hospital, 020125 Bucharest, Romania; 6 Department of Pediatrics, Carol Davila University of Medicine and Pharmacy, 020021 Bucharest, Romania

**Keywords:** Wilson’s disease, hemolytic anemia, laparoscopy, biopsy, cirrhosis, children

## Abstract

Wilson’s disease (WD) is an autosomal recessive disorder, in which the metabolism of copper is affected by metal accumulation in several organs that causes gradual organ degeneration. Since Wilson’s initial description of WD over a century ago, there have been significant improvements in understanding and managing the condition. Nevertheless, the ongoing gap between the onset of symptoms and diagnosis highlights the difficulties in identifying this copper overload disorder early. Despite being a treatable condition, detecting WD early remains a challenge for healthcare professionals at all levels of care, likely due to its rarity. The key challenge is, therefore, to educate physicians on how to identify atypical or infrequent symptoms of WD, prompting them to consider the diagnosis more carefully. The purpose of our review is to draw attention to the difficulties associated with diagnosing pediatric WD, starting from our personal experience of a complex case and then examining relevant literature. In summary, the diagnosis of WD in children is intricate and requires a heightened level of suspicion to identify this infrequent condition. A thorough evaluation by a multidisciplinary team of physicians, along with genetic testing, histopathologic examination, and specialized imaging studies, may be necessary to confirm the diagnosis and guide treatment.

## 1. Introduction

Wilson’s disease (WD), or hepatolenticular degeneration, is a rare genetic disorder that causes the accumulation of copper in various organs, most commonly the liver, brain, and eyes. It is caused by mutations in the ATPase beta polypeptide (ATP7B) gene, which encodes a protein that helps transport copper out of the liver and into the bile ducts [1]. As a result of this copper overload, patients with WD can develop a variety of clinical manifestations, including hepatic, neurologic, and psychiatric [1].

The incidence and prevalence of WD in the pediatric population vary depending on the population studied and the methods of diagnosis. The estimated prevalence ranges from 1 in 30,000 to 1 in 100,000 live births [2]. However, WD is more commonly observed in populations with a higher prevalence of consanguineous marriages. The disease is more predominant in countries such as Pakistan, Egypt, and India, where consanguineous marriages are more common. In these regions, the incidence of WD in children may be higher than the global average [3,4,5].

In pediatric patients with WD, liver disease often presents as jaundice, abdominal enlargement, and abnormal liver function. If left untreated, the build-up of copper in the liver can cause progressive damage to liver tissue, leading to cirrhosis [6]. Cirrhosis leads to a range of symptoms, including fatigue, itching, abdominal pain, and weight loss. In severe cases, cirrhosis can result in liver failure, which can be life-threatening, leading to liver transplant [7].

Diagnosing pediatric WD can be challenging for a variety of reasons. Thus, further innovative markers should be included in the diagnostic strategies [8]. First, children may present with different symptoms than adults, such as failure to thrive, developmental delay, and behavioral changes, which can be easily misinterpreted as other conditions. Second, the onset of symptoms can be insidious, with some children remaining asymptomatic for years, making it difficult to link symptoms to WD. Third, many of the diagnostic tests used to identify WD in adults, such as liver biopsy and serum ceruloplasmin levels, are not as reliable in children due to their immature immune systems and varying growth rates [9]. Additionally, the liver biopsy may be difficult to perform since it poses a significant risk in children [10].

Missed or delayed diagnoses can occur due to healthcare providers’ lack of awareness of this rare condition, resulting in challenges in diagnosing pediatric WD. Thus, there is a need for increased awareness, improved diagnostic tools, and specialized training for healthcare professionals to address these issues.

Therefore, our aim is to present in this review the current knowledge regarding the challenges underlying pediatric WD’s diagnosis, taking as a starting point our personal experience in diagnosing a difficult case.

## 2. Case Presentation

We present the case of a 14-year-old girl, admitted to our hospital for severe anemia and ascites. Regarding her past medical records, she presented with partially investigated hepatic cytolysis (serum aminotransferases repeatedly two to three times normal) during the last four years, as well as severe hemolytic anemia one year before, which required several blood transfusions. However, no viral, autoimmune, or metabolic disorders were discovered to explain her symptoms.

Upon admission to our clinic, she presented with generalized itching, jaundice, and diffuse edema that was palpable in the ankles and pretibial areas of her legs, as well as an enlarged abdomen, indicating poor overall health. During mild palpation, she reported modest pain, and her regular bowel habits in terms of color and consistency were noted. However, the abdomen’s diameter measured around 70 cm due to distention, making it difficult to determine the liver’s diameter, although the spleen was palpable 3–4 cm below the costal margin. Additionally, low puberty development was observed, with the patient having Tanner 1 on the Pubic hair score and Tanner 2 on the Breast Development Scale, as per Tanner’s score [11].

The initial investigation revealed severe anemia (hemoglobin = 3.3 g/dL) with low iron and ferritin levels, without hemolytic elements (high level of conjugated bilirubin = 1.37, low levels of iron level = 16.19 µg/dL and ferritin level = 7.75 µg/dL, normal lactate dehydrogenase, negative Coombs tests), elevated aminotransferases (alanine aminotransaminase—ALT = 98.6 U/L, aspartate aminotransferase—AST = 116.2 U/L), and cholestatic syndrome.

**Figure 1:**
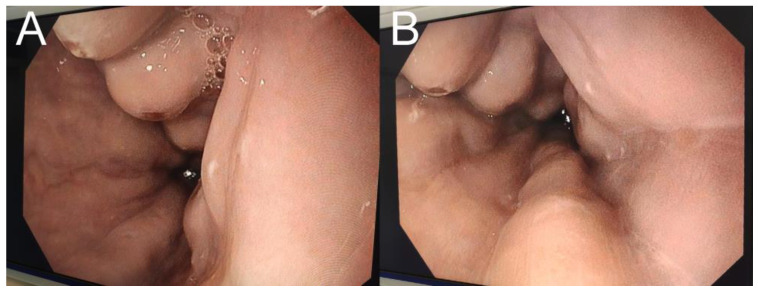
Upper endoscopy revealing grade two esophageal varices. (**A**,**B**) figures reveal tortuous, enlarged blood vessels that occupy less than one-third of the lumen of the esophagus. Some of the varices also present ulcerations.

The abdominal ultrasound (US) identified ascites, minimum hepatomegaly, with relatively homogeneous echogenicity and no other lesion, and homogeneous splenomegaly (17/6 cm). However, the US gave limited and misleading information. The liver aspect was not suggestive of cirrhosis. Additionally, no dilation of the portal vein was noted and no other signs of portal hypertension. However, upon upper endoscopy, grade 2 esophageal varices were revealed, some of them presenting with ulcers (Figure 1).

When all the viral markers (hepatitis B, C, and A virus, Epstein Barr virus, human immunodeficiency virus, Cytomegalovirus), autoimmune hepatitis antibodies (antinuclear, anti-smooth muscle, perinuclear anti-neutrophil cytoplasmic, anti-soluble liver antigen/liver-pancreas, anti-liver kidney microsome type 1 and 3, anti-liver cytosol type 1 antibodies), sclerosing cholangitis antibodies (antineutrophil cytoplasmic, anti-endothelial cell, anti-mitochondrial antibodies), and magnetic resonance cholangiopancreatography came back negative, an extensive differential diagnosis was necessary and a multidisciplinary team was formed.

**Figure 2:**
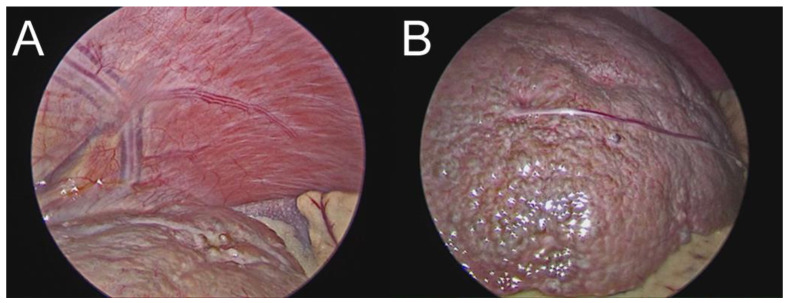
Laparoscopic examination of the liver. (**A**) This image reveals the dilated and distorted blood vessels suggestive for collateral vascularization, while (**B**) depicts the nodular and irregular surface of the liver, with a pale color specific for cirrhosis.

**Figure 3:**
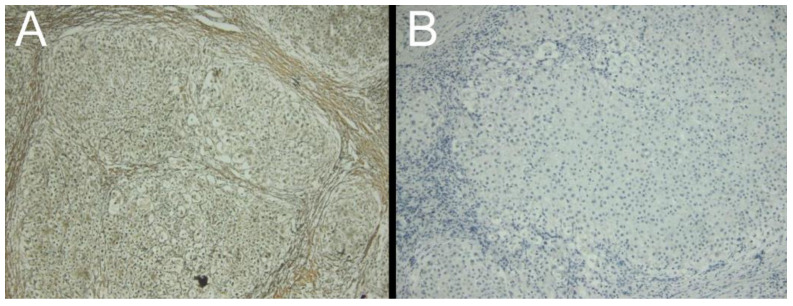
Liver biopsy from our patient presenting with cirrhosis secondary to previously unrecognized Wilson’s disease. (**A**): Gomori stain of hepatic tissue showing copper deposits; (**B**): Rhodanine stain of hepatic tissue showing copper deposits.

WD was suspected when slight changes in the markers for the copper metabolism were noted. The patient had normal ceruloplasmin levels, but upper-normal urinary copper levels, and slightly elevated cupremia (refer to Table 1). Thus, we looked for other signs and symptoms related to the condition. Although the patient did not exhibit neurological symptoms or a Kayser-Fleischer corneal ring, cerebral IRM revealed high T1 signal intensity in the globus pallidus (Figure 2). Moreover, the patient was diagnosed with irritability and depressive adaptive disorder following psychiatric assessment. After performing the D-penicillamine challenge test, urine copper levels increased from 58.86 µg/24 h to 858.00 µg/24 h. WD diagnosis is based on a scoring system [12]; thus, we performed a laparoscopic examination with liver biopsy to identify any disease-specific abnormalities on the liver surface and obtain liver samples. No specific anesthesia or surgery-related complications were observed. The biopsy revealed a nodular liver surface, possibly caused by cirrhosis, as well as splenomegaly and collateral vascularization (Figure 2). Additionally, rhodanine and Gomori colorations of liver samples revealed intracytoplasmic granules specific for copper deposits, suggestive for WD (Figure 3).

Finally, WD diagnosis was established based on a scoring system [12] describing a positive penicillamine challenge test, psychiatric symptoms, but also a high T1 signal intensity in the globus pallidus, and histological liver copper deposits, scoring a total of 5 points, highly suggestive for this disease (Table 2).

**Figure 4:**
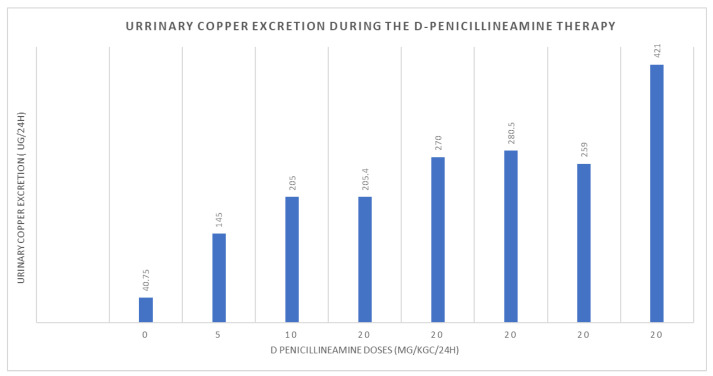
Urinary excretion of copper during the administration of copper chelators therapy.

The patient was immediately put on a low-copper diet and prescribed D-penicillamine, with regular clinical and paraclinical monitoring. Figure 4 illustrates the urinary excretion of copper during the early stages of copper chelator therapy administration. While the patient’s liver enzyme and bilirubin levels remained at the upper limit of normal due to cirrhosis, her overall well-being improved, with no further occurrences of severe anemia or ascites. Furthermore, her puberty development improved and she began to gain weight (increasing her body mass index from 15.8 kg/m^2^ to 19 kg/m^2^).

## 3. Discussion and Literature Review

WD is a genetic disorder, autosomal recessive, characterized by copper accumulation in different organs causing gradual degradation [12]. The cause is an ATP7B mutation, a gene located on chromosome 13, which encodes a copper transport protein. This results in decreased or missing function of the ATP7B transporter necessary for copper integration into apocerulo-plasmin and for biliary excretion of copper [12,13]. This reduction in the primary pathway of hepatic copper removal is responsible for the clinical symptoms and pathology associated with WD. More than 300 ATP7B mutations have been found to be linked to this disease [14]. Some mutations may be more common in certain populations, making them harder to identify in others. Thus, being a genetic disorder, family history plays a critical role in the diagnosis. However, in some cases, there may not be a family history of the disease or the disease may be caused by a de novo mutation [15]. In these cases, a thorough clinical evaluation and genetic testing may be necessary to confirm the diagnosis.

WD has a worldwide distribution, with a prevalence of 1 case per 30,000 live births in most regions [1]. However, population screening by molecular sequencing in the United Kingdom suggested that the prevalence may be as high as 1 case in 7021 [16]. In isolated places, for example, a village on the island of Crete, the prevalence was much higher due to high rates of consanguinity. WD was diagnosed in 1 in 15 births in that village [17]. In some populations, approximately one person in 90 carries an abnormal copy of the ATP7B gene, assuming a prevalence of 1 in 10,000 to 30,000. However, a study of a large French cohort found that approximately one person in 31 is a heterozygous carrier, corresponding to an expected disease prevalence of one case in 1000 live births [18]. The incomplete penetrance of the disease-specific mutations requires further investigation [19]. Although males and females are equally affected by this disease, females are more likely to develop acute liver failure as a consequence [20,21,22].

WD is usually diagnosed between the ages of 5 and 35, but cases have been reported in younger or older patients. Typically, it is diagnosed in pediatric patients around 13 years of age [23]. A study of 143 children with WD found that 15% presented with symptoms or abnormal liver function tests before the age of five [12]. Cases of earlier onset have been reported, with the youngest documented case in the literature being a two-year-old individual [24,25,26,27].

In children, WD makes up around 8 to 10 percent of cases of chronic active hepatitis [28]. The liver disease typically advances without symptoms in most patients, often until adolescence or later, when cirrhosis or acute liver failure complications may arise. In some cases, neurological or psychiatric symptoms occur before the onset of liver disease. Children are more likely to display liver-related symptoms, while neurological symptoms are more common in adolescent and adult patients. Patients who present with neurological symptoms usually do so between the ages of 15 and 21 [29,30,31,32].

According to a study conducted by Manolaki and her colleagues, out of a total of 57 patients with WD, the majority were asymptomatic (40 out of 57), while only 20 had symptoms. Furthermore, among the symptomatic group, only two patients were diagnosed with acute fulminant hepatitis [33]. The literature suggests that individuals with WD tend to develop cirrhosis at a young age. However, despite this, they have a relatively good prognosis [34]. In a tertiary center in Bangladesh, from a group of 60 patients diagnosed with WD, 42 presented with hepatic symptoms, of which 11 (22%) had acute hepatitis, 13 (26%) had acute liver failure, and eight (16%) had chronic liver disease with portal hypertension [35].

Class A Child-Pugh cirrhosis appears to be most commonly described in WD patients, which also predicts favorable outcomes [34]. However, Child-Pugh A cirrhosis is present in less than 1% of WD patients [12]. Diagnosing pediatric cirrhosis due to WD requires a combination of clinical evaluation, laboratory testing, imaging studies, and liver biopsy. Early detection is crucial, as prompt treatment can prevent or delay the progression of liver disease and improve long-term outcomes. Pediatric cirrhosis due to WD is a serious and potentially life-threatening condition that can have significant long-term consequences if not treated promptly [36]. It is important for healthcare providers to be aware of the possibility of WD in children with liver disease and to consider this diagnosis in their evaluation. Early detection and treatment can improve outcomes and prevent or delay the development of cirrhosis.

The manifestations of WD have a broad spectrum, the most common of which is liver, neurologic and ocular disorders, but kidney, psychiatric, cardiac, or endocrine manifestation should also be investigated [2]. Pediatric patients with liver involvement are often asymptomatic [12]. However, they may have mildly elevated liver enzymes, or have clinical signs similar to acute hepatitis, or chronic liver disease, such as compensated or decompensated cirrhosis. Acute liver failure has also been described [12,25,37].

During childhood, neurological symptoms typically occur after the hepatic symptomatology, usually in the third decade of life [13]. The percentage of patients who experience an initial neurological presentation ranges from 18 to 68% [36]. If these neurological symptoms occur before the onset of liver disease, the diagnosis can be even more challenging.

Neurological manifestations are common in pediatric WD and can range from mild symptoms to severe, life-threatening complications. The most prevalent neurological symptoms include ataxia, tremor, dystonia, and parkinsonism, every one of which is frequently accompanied by dysarthria, abnormalities of gait and posture, drooling, and dysphagia [12,13,36]. More than 90% of patients with a neurological presentation have Kayser-Fleischer rings [12,13,35,36,38], but less than 50% of cases show a hepatological presentation [12,13,24,38]. In our case, the Kayser-Fleischer corneal ring and neurological symptoms were absent, making the diagnosis difficult to establish. However, the cerebral IRM showed a strong T1 signal in the globus pallidus. This image appears in WD patients with cirrhosis and portosystemic shunting, which is thought to be the result of manganese buildup [36,39].

Another atypical presentation of Wilson disease in children is that of psychiatric symptoms, such as depression, anxiety, or behavioral changes. These symptoms may occur in the absence of liver or neurological symptoms and may be attributed to other psychiatric conditions, such as bipolar disorder or attention-deficit/hyperactivity disorder [40].

One of the main challenges in diagnosing WD in children is the fact that its symptoms can be vague and nonspecific, making it difficult to recognize. Early symptoms of WD in children can include fatigue, weakness, abdominal pain, and jaundice, which can be mistaken for other more common conditions. An accurate final diagnosis of WD can be made utilizing a diagnostic grading system based on symptoms, biochemical assays evaluating copper metabolism, and molecular testing for ATP7B gene mutations [12].

For example, our patient had a history of severe acute hemolytic Coombs-negative anemia. Thus, in addition to the classical symptoms, some patients with WD may also develop hemolytic anemia, a condition in which red blood cells are destroyed at a faster rate than they are produced. Hemolytic anemia in WD is thought to be caused by the direct toxic effects of copper on red blood cells, leading to oxidative damage and destruction of the cell membrane [25,41,42,43,44]. However, the specific mechanisms are not completely understood [25,41,42,45]. The severity of hemolytic anemia in WD can vary widely, with some patients experiencing mild symptoms, while others may require blood transfusions or other interventions. Up to 17% of WD patients experience hemolytic anemia at some point over the course of the illness, but it is rare for it to manifest as the first symptom [45]. A study from Brazil described only one of 28 pediatric WD patients with hemolytic anemia [44]. Another series of 58 patients described by Hogland et al. had only one presentation with hemolytic anemia [43]. Another illustration is from research done in Australia, where only 22 out of 321 individuals experienced hemolytic anemia [42]. A possible female to male predominance was noticed in the same study, with a prevalence of 15 to 7 (69%) [42]. Symptoms of hemolytic anemia can include fatigue, weakness, pale skin, jaundice, and an enlarged spleen. Diagnosing hemolytic anemia in WD requires a thorough evaluation of the patient’s medical history, physical examination, and laboratory tests. Treatment options for hemolytic anemia in WD may include, besides medications to reduce copper levels in the body, blood transfusions or, in severe cases, splenectomy [45,46,47]. Overall, while hemolytic anemia is not a common complication of WD, it can be a serious and potentially life-threatening condition. As such, it is important for healthcare providers to be aware of the possibility of hemolytic anemia in patients with WD and to monitor patients closely for the development of this condition.

Additionally, the presentation of WD as acute liver failure poses significant diagnostic challenge, as in our case. This is because acute liver failure is a rare but potentially life-threatening condition that requires urgent medical attention. It is not typically associated with this disease, having a prevalence ranging from 4 to 36.5% [48,49,50]. Moreover, the clinical features of acute liver failure can be non-specific and overlap with those of other liver diseases, making it difficult to establish a definitive diagnosis [51]. One of the challenges in diagnosing WD in cases of acute liver failure is that the characteristic features of the disease, such as neurological and psychiatric symptoms, may not be present or may be overshadowed by the severity of the liver failure [52]. As a result, the diagnosis may be delayed or missed, leading to a poor outcome.

Another challenge is that the laboratory investigations used to diagnose WD, such as serum ceruloplasmin levels, may be unreliable in cases of acute liver failure. Ceruloplasmin is an acute-phase reactant and its levels may be elevated in response to liver injury, making it difficult to interpret the results [53]. Approximately 5–20% of cases can have normal levels of ceruloplasmin due to elevation in response to inflammation or infection [54]. Moreover, some mutations in the ATP7B gene can lead to a milder phenotype of WD with normal or only slightly decreased ceruloplasmin levels [55]. In these cases, other laboratory investigations, such as serum copper and urine copper levels, may be more helpful in establishing the diagnosis. Finally, other conditions, such as autoimmune hepatitis, cholestasis, and chronic liver disease, can also cause normal ceruloplasmin levels, leading to a misdiagnosis or delay in diagnosis of WD.

Another challenge in children is that many of the diagnostic tests used in adults, such as liver function tests and serum ceruloplasmin levels, may not be reliable in children. This is because children may have lower levels of ceruloplasmin and may not have developed significant liver damage at the time of diagnosis. In addition, WD can present with a variety of neurological symptoms, such as tremors, dystonia, and psychiatric symptoms, which may not be apparent in young children [29,31]. Diagnosis of WD in children with neurological symptoms requires a high index of suspicion and a thorough evaluation by a pediatric neurologist. Additionally, WD is an autosomal recessive disorder, which means that both parents must carry a copy of the defective gene for a child to develop the disease. This can make the diagnosis of WD more challenging in families with no prior history of the disease.

Pediatric WD can be challenging to diagnose due to its rarity and the wide range of symptoms that can be attributed to other conditions. Therefore, it is essential to use a combination of tests to arrive at an accurate diagnosis, including blood tests, urine tests, and imaging studies. However, laparoscopy with biopsy is one of the most critical diagnostic procedures for pediatric WD [56,57]. During laparoscopy, the surgeon can directly visualize the liver and assess its appearance, size, and texture, as well as obtain samples for biopsy. Liver biopsy is an essential diagnostic tool for pediatric WD because it can confirm the presence of copper accumulation and help determine the extent of liver damage [58,59]. The biopsy sample can also help rule out other liver diseases and provide critical information for determining the best course of treatment. Additionally, laparoscopy with biopsy is a safe and effective procedure for pediatric patients. It is minimally invasive and does not require a large incision, which reduces the risk of complications and shortens recovery time [60]. Laparoscopy with biopsy also allows for a more accurate diagnosis than other non-invasive tests and helps to prevent the unnecessary use of medications or treatments that could be harmful or ineffective.

Liver biopsy involves taking a small tissue sample from the liver and examining it under a microscope. The biopsy sample can confirm the presence of copper accumulation in the liver and assess the extent of liver damage. It can also provide important information about other liver conditions that may have similar symptoms, and guide treatment decisions. Colorations, such as rhodanine and rubeanic acid stains, are commonly used to detect copper accumulation in liver biopsy samples. Rhodanine stains copper-containing structures orange-red, while rubeanic acid stains them black. These stains are highly specific for copper and can identify copper accumulation even in small amounts. The pattern and distribution of copper accumulation in the liver can vary in pediatric WD. For example, copper can accumulate predominantly in the liver lobules or in the portal areas of the liver [60,61,62]. The pattern and distribution of copper accumulation can provide important information about the severity of the disease and the potential for complications. Moreover, biopsy and colorations can help in the diagnosis of other liver conditions that can occur concurrently with WD. These conditions include autoimmune hepatitis, alpha-1 antitrypsin deficiency, and non-alcoholic fatty liver disease [63]. Identifying these conditions can impact the treatment approach and improve outcomes for pediatric patients.

Therefore, biopsy and colorations are important diagnostic tools in pediatric WD. Biopsy samples can confirm the presence of copper accumulation and assess the extent of liver damage, while colorations can identify copper accumulation even in small amounts. These tools can provide crucial information for the accurate diagnosis, assessment of severity, and management of pediatric WD, and help identify other liver conditions that can impact treatment decisions. However, liver biopsy is an invasive procedure that carries some risks, including bleeding, infection, and damage to nearby organs. Therefore, the decision to perform a liver biopsy in pediatric patients with suspected WD should be made by a team of specialists, taking into account the potential benefits and risks for each individual patient.

Treatment for pediatric WD typically involves medications to lower the amount of copper in the body and to prevent further copper accumulation. The primary medication used in the treatment of WD is a chelating agent called D-penicillamine. This agent binds to excess copper in the body and helps to eliminate it through urine. Another medication, trientine, can also be used as a chelating agent [13,56,64]. In addition to medication, a low-copper diet may be recommended. Foods high in copper, such as liver, shellfish, nuts, chocolate, and mushrooms, should be avoided [65]. Once copper levels are stabilized, the maintenance phase begins, which often includes lifelong treatment with zinc supplements to prevent further copper accumulation [64,66]. Propranolol may sometimes be useful for the treatment of cirrhosis-associated portal hypertension [67]. Vitamins, particularly vitamin E and vitamin C, have also been studied as potential treatments for Wilson disease. Vitamin E has been found to have antioxidant properties that may help to reduce oxidative stress in patients with Wilson disease. Vitamin C has also been studied as a potential treatment, as it may help to increase the excretion of copper in the urine [68]. Additionally, vitamins A, D, and K may be useful for treating the deficiencies caused by WD-associated cirrhosis.

Untreated, WD can be fatal. A liver transplant is considered for people who have acute liver failure, are unresponsive to medical therapy, and have end-stage liver disease [13]. Liver transplant can cure WD, but it is a major surgery with potential risks and complications.

WD is a chronic condition that requires lifelong management. Children with WD require ongoing monitoring and treatment to manage their symptoms and prevent complications. Close collaboration with a team of healthcare providers and specialists is necessary to provide optimal care for children with WD.

## 4. Conclusions

In conclusion, diagnosing WD in children can be particularly challenging due to its rare non-specific symptoms, lack of awareness, genetic variability, atypical presentations, and the need for appropriate diagnostic tests. Pediatricians and other healthcare providers should be aware of the signs and symptoms of this disease, such as unexplained liver disease, neurological symptoms, and Kayser-Fleischer rings, and consider genetic testing or liver biopsies in children with a suspected diagnosis. Early detection and management of WD are critical to prevent long-term complications and improve outcomes. A multidisciplinary approach, involving pediatric hepatologists, neurologists, geneticists, nutritionists, pediatric surgeons, and anesthesiologists is essential for the optimal care and management of children with WD.

## Figures and Tables

**Table 1:** Copper metabolism test results.

Test	Results	References
Ceruloplasmin	0.469	0.20–0.60 g/L
Urinary copper/24 h	58.86	<60 ug/24h
Serum copper level	193	90–190 ug/dL

**Table 2:** The diagnostic score for Wilson’s disease. Modified after the consensus agreement [12].

Score	−1	0	1	2	4
**Keyser-Fleischer rings**		Absent		Present	
**Neuropsychiatric symptoms suggestive of WD (or typical brain MRI)**		Absent		Present	
**Coombs negative hemolytic anemia+ high serum copper**		Absent	Present		
**Urinary copper (in the absence of acute hepatitis)**		Normal	1–2× ULN	>2× ULN or normal but >5× ULN 1 day after challenge with 2 × 0.5 g D-penicillamine	
**Liver copper quantitative**	Normal		<5× ULN (<250 ug/g)	>5× ULN (>250 ug/g)	
**Rhodanine positive hepatocytes**		Absent	Present		
**Serum ceruloplasmin (nephelometric assay)**		>0.2 g/L	0.1–0.2 g/L	<0.1 g/L	
**Disease-causing mutations detected**		None	1		2
**0–1: Unlikely**		2–3: Probable		4 or more:	High likely

Abbreviations: WD, Wilson Disease; ULN, Upper limit normal. The colored values represent the criteria found in our patient that sum the total number of 5 which means a high possibility of Wilson disease.

## Data Availability

No new data were created or analyzed in this study. Data sharing is not applicable to this article.
